# Amorphous calcium magnesium phosphate nanocomposites with superior osteogenic activity for bone regeneration

**DOI:** 10.1093/rb/rbab068

**Published:** 2021-11-24

**Authors:** Yingying Jiang, Shuo Tan, Jianping Hu, Xin Chen, Feng Chen, Qianting Yao, Zhi Zhou, Xiansong Wang, Zifei Zhou, Yunshan Fan, Junjian Liu, Yize Lin, Lijia Liu, Shisheng He

**Affiliations:** 1 Department of Orthopedic, Spinal Pain Research Institute, Shanghai Tenth People’s Hospital, Tongji University School of Medicine, Shanghai 200072, China; 2 Institute of Translational Medicine, Shanghai University, Shanghai 200444, China; 3 National Engineering Research Center for Nanotechnology, Shanghai 200241, China; 4 Institute of Functional Nano & Soft Materials (FUNSOM), Jiangsu Key Laboratory for Carbon-Based Functional Materials & Devices, Soochow University, Suzhou 215123, China; 5 Shanghai Key Laboratory of Tissue Engineering, Shanghai Ninth People's Hospital, Shanghai Jiao Tong University School of Medicine, Shanghai 200011, China

**Keywords:** biomineralization, bone, materials synthesize, biomaterial-cell

## Abstract

The seek of bioactive materials for promoting bone regeneration is a challenging and long-term task. Functionalization with inorganic metal ions or drug molecules is considered effective strategies to improve the bioactivity of various existing biomaterials. Herein, amorphous calcium magnesium phosphate (ACMP) nanoparticles and simvastatin (SIM)-loaded ACMP (ACMP/SIM) nanocomposites were developed via a simple co-precipitation strategy. The physiochemical property of ACMP/SIM was explored using transmission electron microscope (TEM), Fourier transform infrared spectroscopy (FTIR), powder X-ray diffraction (XRD) and high-performance liquid chromatograph (HPLC), and the role of Mg^2+^ in the formation of ACMP/SIM was revealed using X-ray absorption near-edge structure (XANES). After that, the transformation process of ACMP/SIM in simulated body fluid (SBF) was also tracked to simulate and explore the *in vivo* mineralization performance of materials. We find that ACMP/SIM releases ions of Ca^2+^, Mg^2+^ and ${\text{PO}}_{4}^{3-}$, when it is immersed in SBF at 37°C, and a phase transformation occurred during which the initially amorphous ACMP turns into self-assembled hydroxyapatite (HAP). Furthermore, ACMP/SIM displays high cytocompatibility and promotes the proliferation and osteogenic differentiation of MC3T3-E1 cells. For the *in vivo* studies, lamellar ACMP/SIM/Collagen scaffolds with aligned pore structures were prepared and used to repair a rat defect model in calvaria. ACMP/SIM/Collagen scaffolds show a positive effect in promoting the regeneration of calvaria defect after 12 weeks. The bioactive ACMP/SIM nanocomposites are promising as bone repair materials. Considering the facile preparation process and superior *in vitro/vivo* bioactivity, the as-prepared ACMP/SIM would be a potential candidate for bone related biomedical applications.

## Introduction

Bones are complex composites with hierarchical structures which consist of both biomacromolecules and biominerals [1]. The biomacromolecules include type I collagen, non-collagenous proteins (e.g. proteoglycans), minor amounts of lipids and osteogenic factors (e.g. bone morphogenetic proteins (BMPs)) [2, 3]. The formation of bone is rigorously regulated by four types of bone cells, which are osteoblasts, osteoclasts, osteocytes and bone-lining cells. The interactions between the cells and the extracellular biomacromolecule matrix play critical roles in the formation of bone structure [4–6]. The main inorganic component of native bone is calcium phosphate (CaP)-based biominerals with a crystal structure of hydroxyapatite (HAP) [7]. Synthetic CaP-based biomaterials have demonstrated excellent biodegradability, bioactivity and osteoconductivity, and are widely used for bone-defect repair and prevention of bone-loss diseases (e.g. osteoporosis) [8–12]. The detailed composition of CaP in natural bones is far more complicated, since it usually contains other ions that substitute the original CaP structure such as ${\text{CO}}_{3}^{2}$^−^, Mg^2+^ and Na^+^ [2]. Mg^2+^ ions are known directly involved in numerous biological metabolisms and regulate bone tissue formation. It has gained great attention for its role in bone regeneration. For example, Liu *et al.* [13] reported that Mg^2+^ ions not only promote the adhesion of bone marrow mesenchymal stem cells (BMSCs) by up-regulating the expression of integrin α5β1 on cell membrane, but also stimulate the osteogenic differentiation and enhance bone regeneration [14].

Bone regeneration is a multi-stage process, including hemostasis, inflammation and remodeling tissue formation. All these phases are strictly connected and play a key role for a complete and proper restoration of the injured tissue [15]. The completion of the whole process requires the interplay among multiple biomaterials. Angiogenesis remodeling is crucial for bone repair since blood vessels can transport the necessary nutrition [16]. Simvastatin (SIM) was reported to be able to increase the expression of BMP-2 [17] and promote osteoblast differentiation [18] by up-regulating the expression of ostetrix and osteocalcin (OCN) genes [19] and via the membrane-bound Ras/Smad/Erk/BMP-2 pathway [20]. Furthermore, SIM also can enhance angiogenesis of tissue via the up-regulation of vascular endothelial growth factor (VEGF) in dose-dependent manners [21, 22]. This property is of particular value for bone regeneration application. Previous research also has demonstrated that SIM can be used to functionalize biomaterials to achieve the enhanced neovascularization [23].

There are some reports focusing on SIM together with CaP cements or scaffolds for bone regeneration [24–27], osteoporosis therapy [28–31] and dental rehabilitation [32], due to the inhibition of osteoclastic mediated bone resorption and up-regulation of angiopoiesis. Magnesium alloys deposited with SIM have also been investigated for bone repair with a controlled release of SIM [33, 34]. Magnesium alloys deposited with SIM have also been investigated for bone repair with a controlled release of SIM [35]. However, the osteogenic activity of SIM incorporated amorphous calcium magnesium phosphate (ACMP) has not been investigated yet.

Herein, a simple co-precipitation strategy has been developed to prepare ACMP nanoparticles and SIM-loaded ACMP (ACMP/SIM) nanocomposites with superior osteogenic activity. Then, the method of X-ray absorption near-edge structure (XANES) has been first used to investigate the influence of Mg^2+^ ions on the formation of ACMP. Moreover, the transformation process of ACMP/SIM in SBF was also tracked to reveal the mineralization performance and the mechanism in the transformation of samples from ACMP to HAP. Furthermore, the high cytocompatibility and bioactivity of ACMP/SIM in promoting osteogenic differentiation have been proved by coculture with MC3T3-E1 cells. Finally, lamellar scaffolds of ACMP/SIM/Collagen with aligned pore structure were prepared for the study of *in vivo* bone-defect repair, which clearly showed a positive effect in promoting bone regeneration of calvaria defect.

## Materials and methods

### Chemicals

Calcium chloride (CaCl_2_), sodium chloride (NaCl) and hydrochloric acid (HCl, 36.0–8.0%) were purchased from Sinopharm Chemical Reagent Co., Ltd. Sodium phosphate tribasic dodecahydrate (Na_3_PO_4_·12H_2_O), sodium bicarbonate (NaHCO_3_, 99.8%), potassium chloride (KCl), potassium phosphate dibasic trihydrate (K_2_HPO_4_·3H_2_O), magnesium chloride hexahydrate (MgCl_2_·6H_2_O), sodium sulfate (Na_2_SO_4_) and tris(hydroxymethyl)aminomethane hydrochloride ((HOCH_2_)_3_CNH_2_, 99.0%) were purchased from Aladdin Co., Ltd. SIM was purchased from Shanghai SHFENG biological technology Co., Ltd. Collagens obtained from pigskin were purchased from Kele Biological Technology Co., Ltd, China. The chemicals were used as received without further purification.

### Preparation of calcium magnesium phosphate (ACMP) and SIM-loaded ACMP (ACMP/SIM)

Typically, MgCl_2_·6H_2_O and CaCl_2_ were dissolved in 7 ml of deionized water and mixed with a SIM solution dissolved in 5 ml of acetonitrile to obtain solution A. Then, 25 ml solution B containing Na_3_PO_4_·12H_2_O was slowly added into solution A under magnetic stirring for 10 min. The products were collected by centrifugation, washed at least three times using deionized water, and freeze-dried for further use. The specific amounts of chemical reagents added in the reaction solutions for preparing different samples are given in Table 1.

**Table 1.** Specific parameters for the preparation of ACMP and ACMP/SIM with different contents of Mg^2+^

	Solution A			Solution B
	m (MgCl_2_·6H_2_O)	m (CaCl_2_)	m (SIM)	m (Na_3_PO_4_·12H_2_O)
ACMP-0% (HA Rods)	0 g	0.120 g	0 g	0.670 g
ACMP-10% (ACMP)	0.020 g	0.110 g	0 g	0.670 g
ACMP-50% (ACMP-2)	0.110 g	0.060 g	0 g	0.670 g
ACMP-100% (ACMP-3)	0.220 g	0 g	0 g	0.670 g
ACMP/SIM	0.020 g	0.110 g	0.002 g	0.670 g

### Characterizations

The crystal structure of the samples was determined by an X-ray powder diffractometer (XRD, Bruker advance D8, Germany) supplemented with Rigaku D/max 40 kV and Cu Kα radiation. Scanning electron microscopy (SEM, Hitachi S-4800, Japan) and transmission electron microscopy (TEM, JEOL JEM-2100F, Japan) together with an energy-dispersive spectroscopy (EDS, JED2300) were performed to investigate the microstructures and chemical composition. The concentration of released Ca^2+^, Mg^2+^ and ${\text{PO}}_{4}^{3}$^−^ ions of ACMP/SIM in simulated body fluid (SBF) was analyzed via a ZEEnit^®^700P flame and graphite furnace atomic absorption spectrometer (AAS, Analytik Jena, Germany). The molecular structure and chemical composition of the samples were investigated using a Fourier transform infrared (FTIR) spectrometer (Nicolet iS5, Thermo Scientific, USA).

The content of SIM in ACPC/SIM nanocomposites was measured using a high-performance liquid chromatography (HPLC, Germany) with an Agilent 1260 Infinity Quaternary LC system. Five milligrams of ACMP/SIM were dissolved using 1 ml of 1M HCl solution and then detected by the diode array detector (DAD) at a wavelength of 238 nm. An ODS Hypersil C18 column (4.6 ×250 mm, 5 mm) was used, the mobile phase solution consisted of 81% methanol and 19% distilled water, the flow rate of the mobile phase was 1.0 ml min^−1^, the injection volume was 20 μl and the temperature of the chromatographic column was 25°C. The HPLC chromatograms of a series of SIM ethanol solutions (0, 50, 100, 150, 200 and 250 μg ml^−1^) were also detected to obtain the absorbance-concentration curve.

Ca, Mg and P K-edge XANES spectra of the samples were obtained at Canadian Light Source (CLS) using a Si (111) crystal monochromator at the Soft X-ray Microcharacterization Beamline (SXRMB), which is operated with an energy range of 1.7–10 keV. The total electron yield (TEY) and X-ray fluorescence yield (FLY) XANES spectra were collected simultaneously to track the surface and bulk sensitivity.

### The transformation of ACMP/SIM in SBF

About 0.030 g of the as-prepared ACMP/SIM nanocomposites was added into 30 ml of SBF solution (pH 7.4) to explore the stability and structural transformation of ACMP/SIM in SBF. The whole process was conducted at 37°C under a constant shaking frequency of 140 rpm. The standard SBF was prepared according to standard protocols [36]. The resultant samples were, respectively, collected at 0.5, 3, 12, 24, 36 and 96 h, and then were washed by deionized water for three times and freeze-dried for further characterization.

### Cell viability tests *in vitro*

MC3T3-E1 cells which derive from pre-osteoblastic cell line were used to evaluate the biocompatibility and bioactivity of the materials in the following experiments. The MC3T3-E1 cells were purchased from Procell Life Science & Technology Co., Ltd. and cultured in an α-minimum essential medium (α-MEM, Cytiva Life Science) supplemented with 10% fetal bovine serum and 1% penicillin–streptomycin under a 5% CO_2_ humidified atmosphere at 37°C. MC3T3-E1 cells were seeded at a concentration of 2 × 10^3^ cells per well in a 96-well plate and cultured with 100 μl α-MEM. The as-prepared HA rods, ACMP and ACMP/SIM powders were sterilized under ultraviolet light for at least 4 h. MC3T3-E1 cells were treated with the sterilized powders at different concentrations. A CCK-8 assay was performed to test the effect of the samples on the viability of MC3T3-E1 cells, after the cells were incubated with samples for 1d, 2d, 3d and 7d. The detailed values were determined using a microplate reader (BioTek Instruments, USA) at a wavelength of 450 nm. The data were presented as the mean value of six parallel measurements.

### Alkaline phosphatase staining

The MC3T3-E1s treated with different samples in 24-well plates were washed with PBS at day 7 and 14. Then paraformaldehyde solution (4%) was added for fixation and then washed with PBS. Alkaline phosphatase (ALP) activity of the sample-free or sample-treated MC3T3-E1s was assessed at day 7 and 14 using an ALP staining kit purchased from Beyotime Biotechnology (Shanghai, China). The optical images were obtained using a Leica inverted microscope. The concentration of HA rods, ACMP and ACMP/SIM for ALP staining test was 100 μg ml^−1^.

### Western blots

The MC3T3-E1s treated with 100 μg ml^−1^ of HA rods, ACMP and ACMP/SIM in a 6-well plate for 7 and 14 days were washed with PBS and collected using an RIPA buffer (containing proteinase and phosphatase inhibitors (Beyotime Biotechnology, Shanghai, China)) at 4°C for 10 min and then centrifuged with a speed of 12 000 rpm at 4°C for 5 min. The supernatants were quantified with a BCA protein quantitative kit (Beyotime Biotechnology, Shanghai, China). The same amounts of protein lysates collected from the aforementioned groups were added to the SDS-PAGE. Proteins were separated via gel electrophoresis and transferred to a nitrocellulose membrane sheet. After being blocked by skim milk, different primary antibodies (anti-osteopontin (1:1000, Abcam, UK), anti-osteocalcin (1:1000, Santa Cruz, USA), and anti-actin (1:1000, Abcam, UK)) were incubated at 4°C overnight. Thereafter, the membranes were washed with PBST (PBS with Tween) and incubated with second antibodies (anti-mouse or anti-rabbit second antibody, 1:3000, CST, USA) at room temperature for 60 min. The Western blot bands were visualized by an electrochemical luminescence solution (LI-COR, USA). HA rods, ACMP and ACMP/SIM (100 μg ml^−1^) were co-cultured with MC3T3-E1s for western blots.

### Real-time quantitative PCR

The total RNA of MC3T3-E1s treated with HA rods, ACMP and ACMP/SIM at a concentration of 100 μg ml^−1^ at day 7 and 14 was extracted using Trizol reagent (Molecular Research Center). The cDNA was generated using the PrimeScriptTM RT reagent Kit with gDNA Eraser (TaKaRa). The expression of OCN, osteopontin (OPN), Osterix, Runx2 and type I collagen was determined by TB Green^®^ Premix Ex Taq™ II (Tli RNaseH Plus) (TaKaRa). The relative gene expression was normalized by the glyceraldehyde-3-phosphate dehydrogenase (GAPDH) gene. Real-time quantitative PCR amplifications were performed using the following primers (Table 2).

**Table 2.** Primers for the real-time quantitative PCR amplifications

Primers	Oligo sequences 5′-3′
Osteocalcin-forward	GCTGCCCTAAAGCCAAACTCT
Osteocalcin-reverse	AGAGGACAGGGAGGATCAAGTTC
Osteopontin-forward	AGCAAGAAACTCTTCCAAGCAA
Osteopontin-reverse	GTGAGATTCGTCAGATTCATCCG
Osterix-forward	ATGGCGTCCTCTCTGCTTG
Osterix-reverse	TGAAAGGTCAGCGTATGGCTT
Runx2-forward	CCGCACGACAACCGCACCAT
Runx2-reverse	CGCTCCGGCCCACAAATCTC
Type I collagen-forward	GCAACAGTCGCTTCACCTACA
Type I collagen-reverse	CAATGTCCAAGGGAGCCACAT
GAPDH-forward	TCTCTGCTCCTCCTGTTCGA
GAPDH-reverse	GCGCCCAATACGACCAAATC

### Fabrication of ACMP/SIM/Collagen scaffold with cancellous structure

The 3D scaffolds of ACMP/SIM/Collagen with cancellous structure are prepared. The methodology for the preparation of the collagen-based scaffold with controlled pore structure was referred to the literature reported by Schoof *et al.* [37]. In brief, 0.086 g of ACMP/SIM powders were dispersed into 10 ml solution containing 0.200 g type I collagen (Kele Biological Technology Co., Ltd, China) via magnetic stirring, then 50 μl of the mixed solution was added into a 96-well plate with a diameter of 5 mm. The plate containing ACMP/SIM/collagen solutions were quickly frozen via unidirectional solidification method using liquid nitrogen, subsequent freeze-drying was followed and then the dried products were stored at −20°C for further use.

### ACMP/SIM/Collagen scaffold for *in vivo* bone-defect repair

Twenty male rats (about 250 g, 7 weeks-old) were purchased from Shanghai SLAC laboratory animal Co., Ltd, China. All the animal experiments were performed according to the protocols approved by Ethics Committee of Shanghai Tenth People’s Hospital. The animal ethical permission number and date are SHDSYY-2019-T0011 and 28 November 2019. All the rats were subjected to specific pathogen free conditions (12 h light/dark cycle, 23°C) and were provided with food and water. After the rats were anesthetized with pentobarbitital solution, the surgery region was shaved and aseptically prepared using iodophor. The skin and periostea were incised by scalpel, and two cylindrical defects with a diameter of 5 mm were created on both sides of the skull symmetrically using a 5 mm trephine. Then, the sterilized cylindrical HA rods/Collagen, ACMP/Collagen and ACMP/SIM/Collagen scaffolds with a diameter of 5 mm and the thickness of 1 mm were sterilized in ultraviolet light for at least 4 h before being used to fill the calvaria bone defects. The direction of aligned pore structure was parallel to the surface of bone. The unfilled calvaria bone defects on the left of 10 rats were set as the blank group. At the end of the surgery, the periostea and skin were sutured. At 8, 12 weeks post-implantation, 5 rats were sacrificed for every group and the calvaria bone were collected and fixed in 4% paraformaldehyde solution for further characterization.

The micro-computed tomography (micro-CT) analysis of the collected calvaria bone was conducted (at least three parallel samples for each time point) using a Micro-CT 80 (Scanco Medical, Switzerland) to characterize their 3D structure and newly formed calvaria bone.

### Histological analysis

The harvested calvaria bone samples were washed by PBS solution and fixed using 4% paraformaldehyde solution. Then, calvaria bones were decalcified in an EDTA solution (0.5 M, pH 8.0) for 1 month and then embedded in paraffin. Histological sections were prepared for the following hematoxylin and eosin (H&E) staining and Masson’s trichrome. All the chemicals are purchased from Wuhan Servicebio technology Co., Ltd, China. The optical images were taken by a Leica inverted microscope.

### Statistical analysis

All the measurements were performed in triplicate and shown as the means ± standard deviation. Comparisons among different groups were evaluated by ANOVA and Student’s *t*-test. Only when the *P*-values is below 0.05, the data were regarded as of statistical significance.

## Results and discussion

### Materials preparation and characterization

**Figure 1.**
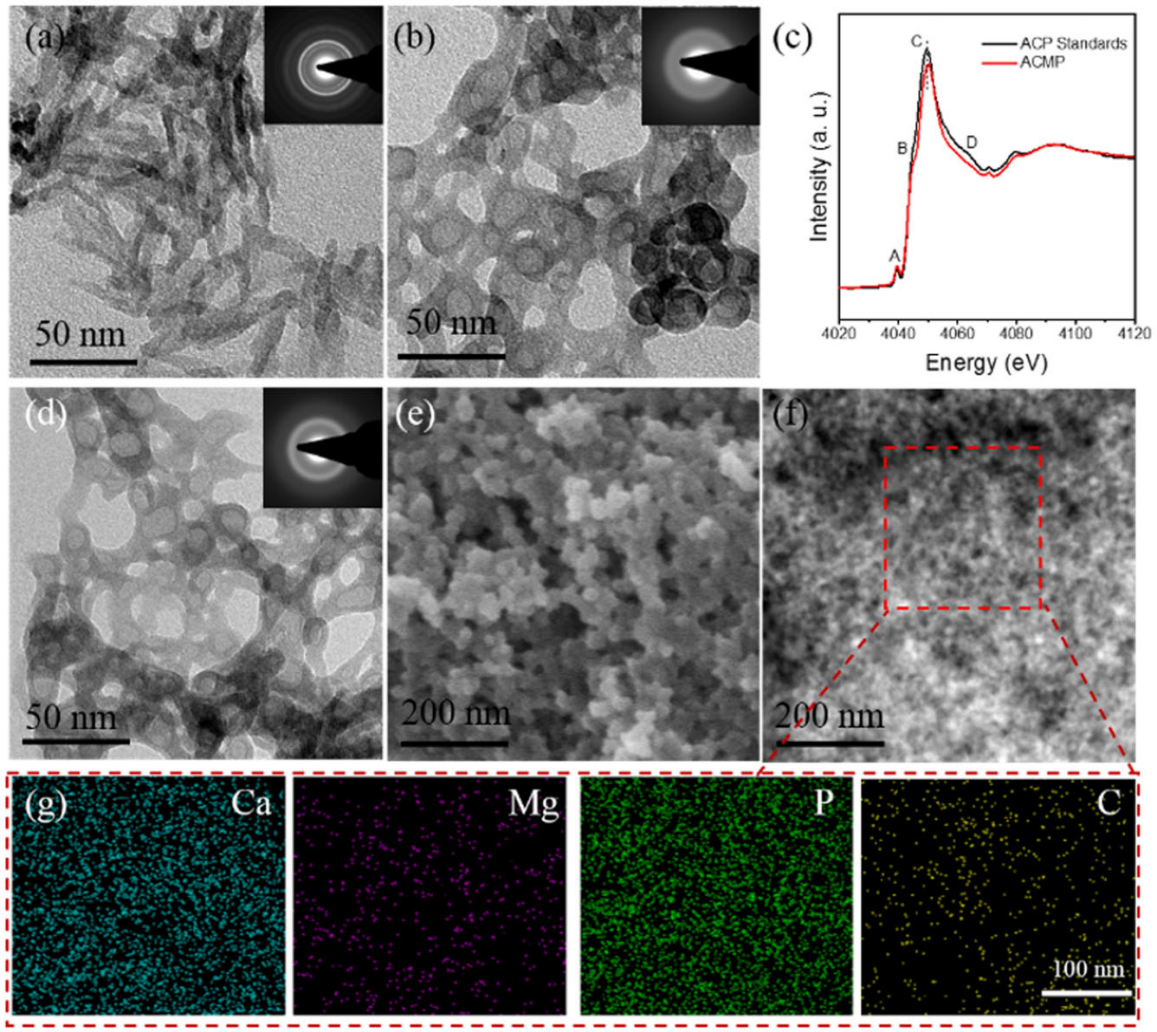
The Characterization of ACMP/SIM prepared under the regulation of Mg^2+^ ions and SIM. TEM images of samples with different Ca/Mg ratios of (**a**) 10:0 (HAP rods) and (**b**) 10:1 (ACMP); (**c**) Ca K-edge XANES spectra of ACP standards and ACMP/SIM; (**d**) TEM, (**e, f**) SEM images of ACMP/SIM nanocomposites; (**g**) the elements mapping of Ca, Mg, P and C in ACMP/SIM nanocomposites, indicating the uniform distribution of Ca^2+^, Mg^2+^, ${\text{PO}}_{4}^{3-}$ and SIM. The inserts (a, b and d) are SAED patterns of samples. The TEM images of (a) and (b) represent typical nanorod structure of HAP and amorphous structure of ACMP, respectively

TEM micrograph (Fig. 1a) of the sample prepared without adding Mg^2+^ indicates a crystalline nanorod structure which is a typical morphology of HAP. Meanwhile, when Mg^2+^ ions were added into the reaction solution (Fig. 1b, Mg/Ca = 1:10), the resultant samples of ACMP display a significantly different nanoparticle structure with a diameter of ∼30 nm, and the SAED pattern of the ACMP nanoparticle show a typical amorphous phase. Supplementary Fig. S1 gives the TEM micrographs and SAED patterns of ACMP-2 (Mg/Ca = 5:5) and ACMP-3 (Mg/Ca = 10:0) samples, and the both products exhibit similar morphology and amorphous phases with ACMP. To further explain the influence of Mg^2+^ in the formation of ACMP, XANES spectra of ACMP and ACP standards were collected and shown in Fig. 1c and Supplementary Fig. S5. The XANES spectra at the Ca K-edge of ACMP/SIM and ACP standards were compared in Fig. 1c, the principal peak ‘C’ of ACMP/SIM moved to a higher energy, that’s due to Mg^2+^ ions substitute some of the positions of Ca^2+^ ions in ACP. Herein, after doping with Mg^2+^ ions, both the morphology and crystal phase of CaP were influenced, so it is extracted that the Mg^2+^ ion plays a significant role on the formation of ACMP nanocomposites. It is reported that Mg^2+^ ions have a positive role in osteogenic differentiation [38], while excessive ion concentration will inhibit the differentiation. In case the released Mg^2+^ ions may be excessive, ACMP with a Mg/Ca ration of 1:10 was explored for further application instead of higher doped ACMP.

Figure 1d–f shows the TEM, SEM and STEM micrographs of SIM-loaded ACMP (ACMP/SIM) nanocomposites. Comparing with ACMP, ACMP/SIM nanocomposite which has been prepared with same molar ratio of Ca^2+^, Mg^2+^ and ${\text{PO}}_{4}^{3}$^−^ ions almost displays the same structure with ACMP. STEM micrograph shows that ACMP/SIM nanoparticles with diameters of about 30 nm consist of much smaller particles. Figure 1f shows the element mappings of Ca, Mg, P and C in ACMP/SIM nanocomposite, and the STEM pictures displays the uniform distribution of C and Mg in ACMP/SIM nanocomposites. The distribution of C matches the particle shape with smaller size shown in Fig. 1f, the atomic percentages of ACMP-0, ACMP, ACMP-2, ACMP-3 and ACMP/SIM are given in Supplementary Table S1, the data indicate that the composition of different samples follows the stoichiometry of precursor ions, i.e. the content of Mg^2+^ in ACMP increased with the input of Mg^2+^ in the precursor solutions. Additionally, the TEM micrographs of ACMP and ACMP/SIM displayed hollow structure, while the corresponding STEM micrographs indicated that ACMP/SIM nanoparticles were solid, so the displayed hollow structures shown in TEM images were due to instrumental reasons.

**Figure 2.**
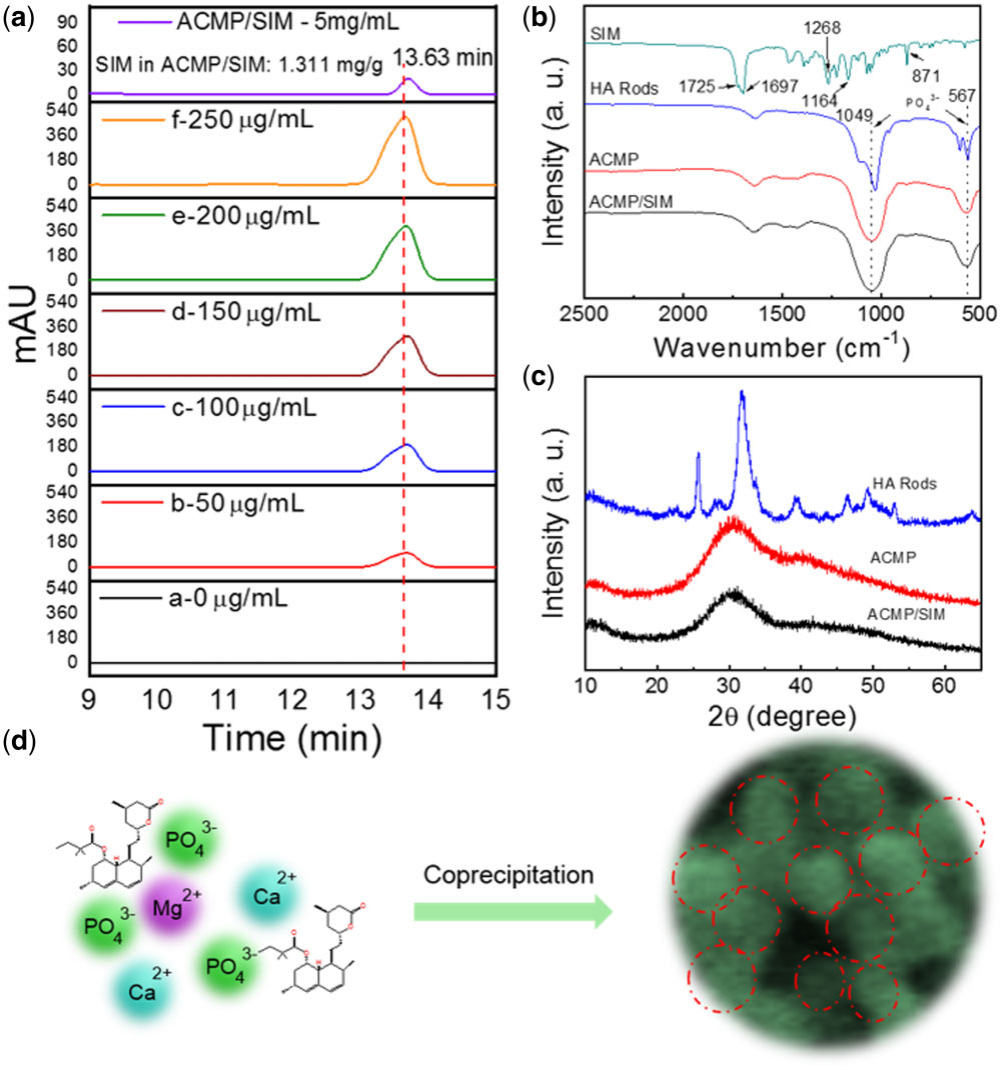
Formation illustration of ACMP/SIM prepared under the regulation of Mg^2+^ ions and SIM. (**a**) HPLC chromatogram of a series of SIM solutions with different concentrations (0, 50, 100, 150, 200, 250 μg ml^−1^) and dissolved ACMP/SIM solution (5 mg ml^−1^), showing the content of incorporated SIM in ACMP/SIM is 1.311 mg/g; (**b**) FTIR spectra and (c) XRD patterns of ACMP, ACMP/SIM and HA rods; (**d**) The proposed formation process of the simvastatin and magnesium incorporated calcium phosphate nanocomposites (ACMP/SIM) with a Ca/Mg ratio of 10:1

To further analyze the content of SIM in the nanocomposites, the as-prepared ACMP/SIM was dissolved in HCl solution and analyzed via HPLC. Figure 2a shows the HPLC chromatogram of a series of SIM solutions and the dissolved ACMP/SIM solution (5 mg ml^−1^), the remaining time of SIM is 13.63 min, and the related peak area-concentration curve obtained from HPLC chromatogram is shown in Supplementary Fig. S2. The small peak in the HPLC chromatogram of ACMP/SIM solution clearly reveals the existence of SIM in the nanocomposites and the content is 1.311 mg g^−1^. The release kinetics of SIM were also tracked using HPLC, while the concentration of SIM was too low and remained undetectable in the released solution due to the strong hydrophobicity [39]. Figure 2b shows the FTIR spectra of SIM and the prepared samples, the FTIR spectrum of ACMP/SIM did not obviously show the characteristic peaks of SIM, which is due to the low content of SIM in ACMP/SIM. The intense absorption peaks at about 1049 and 567 cm^−1^ were attributed to ${\text{PO}}_{4}^{3}$^−^ group, and the peaks of HA rods split into two peaks, which is the feature of crystallinity apatite phase. The XRD patterns in Fig. 2c also indicate the crystalline apatite phase of HA Rods, and amorphous phase of ACMP and ACMP/SIM. Figure 2d shows the proposed formation process of the ACMP/SIM with a Ca/Mg ratio of 10:1. Mg^2+^ ions took over certain sites of Ca^2+^ ions and hindered the crystallization of HAP, meanwhile, SIM was physically adsorbed onto the nanoparticles and simultaneously loaded on the amorphous ACMP.

### Stability and structural transformation of ACMP/SIM in SBF

**Figure 3.**
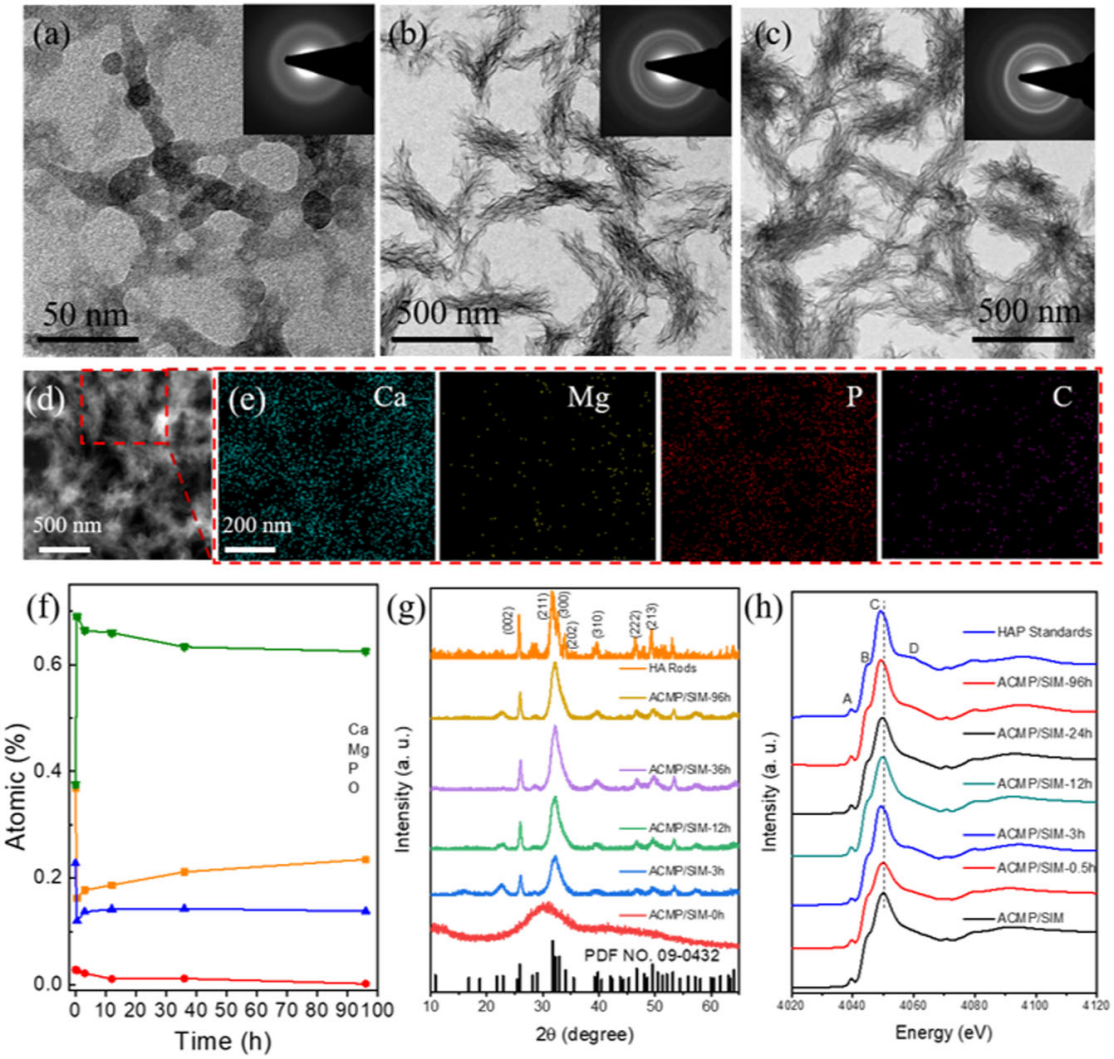
Characterization of the stability and phase transformation of ACMP/SIM in SBF. TEM images of the simvastatin and magnesium incorporated calcium phosphate nanocomposites (ACMP/SIM) with a Ca/Mg ratio of 10:1 dispersed in simulated body fluid (SBF) for (**a**) 0.5 h, (**b**) 3 h, (**c**) 96 h (insert: SAED patterns); (**d**) STEM images of ACMP/SIM dispersed in SBF for 96 h; (**e**) The Ca, Mg, P, O and C distribution mappings of selected area in Fig. 3d; (**f**) The atomic percentage of Ca, Mg, P and O in ACMP/SIM after being dispersed in SBF for different time; (g) XRD patterns of ACMP/SIM after being dispersed in SBF for different time points; (**h**) Ca K-edge XANES spectra of HAP standards, Mg_3_(PO_4_)_2_ standards, and ACMP/SIM samples dispersed in SBF for different time

To investigate the stability and structural transformation of ACMP/SIM, the samples were immersed into SBF at 37°C for different time. As shown in Fig. 3, ACMP/SIM still were nanoparticles with amorphous phase after being immersed in SBF for 0.5 h (Fig. 3a). Thereafter, ACMP/SIM transformed into HAP with a structure of assembled nanoplate bundles in 3 h (Fig. 3b). The resultant HAP nanoplate bundles were stable and did not transform to other structures in the following hours (Fig. 3c and d and Supplementary Fig. S3). Figure 3e and Supplementary Fig. S4 shows the elements distribution of ACMP/SIM after being immersed in SBF for 0.5 and 96 h, and indicates that the resultant minerals consist of much less Mg^2+^ than ACMP/SIM (Fig. 1g). Figure 3f shows the atomic percentage of Ca, Mg, P and O in ACMP/SIM after being treated in SBF for different time. The content of oxygen in ACMP/SIM increased due to the formation of OH groups, while other elements decreased as time went on. ACMP/SIM immersed in SBF quickly release the ions of Ca^2+^ and ${\text{PO}}_{4}^{3}$^−^ in the initial period. While 0.5 h later, the content of Ca^2+^ and ${\text{PO}}_{4}^{3}$^−^ in the samples slightly increased, which possibly indicate the mineralization of ACMP/SIM. However, Mg^2+^ ions in ACMP/SIM decreased obviously in the first 10 h and kept releasing in the following time. Magnesium ions play an important role in maintaining the stability of amorphous CaP. Therefore, the reduction of magnesium ions in this process may play an important role in the transformation of ACMP to HAP, including chemical phase and morphology. The XRD patterns in Fig. 3g reconfirm the phase transformation process, which is identical with the analysis from the SAED patterns.

XANES is an elemental and chemical environment sensitive technology. XRD is not as sensitive as XANES for the analysis of amorphous materials. Herein, XANES spectra of ACMP/SIM and the related minerals have been collected and shown in Fig. 3h and Supplementary Fig. S5, which are used to explore the transformation of ACMP/SIM in SBF. The white line ‘C’ of ACMP/SIM moved to a lower energy gradually with the mineralizing time increasing, which reveals slight changes of surrounding environment and/or unoccupied states of Ca atoms, and the reasonable explanation possibly are the release of Mg^2+^ and/or the mineralization of ACMP/SIM. Mg^2+^ ions moved out of ACMP/SIM, and ACMP/SIM transformed into HAP gradually, meanwhile, XANES spectra of ACMP/SIM-96h at the Ca K-edge showed a post shoulder close to main peak ‘C’, which is due to the occurrence of two different Ca atoms in HAP. It’s clear that the results obtained from XANES spectra are in great agreement with that obtained from TEM micrographs, FTIR spectra and XRD patterns.

Hence, from the characterization above, it is clear that Mg^2+^ ions influenced the crystallization process of HAP, since crystalline HAP nanorods were obtained when Mg^2+^ ions were absent from the co-precipitation of Ca^2+^ and ${\text{PO}}_{4}^{3}$^−^, while an amorphous structure of ACMP was obtained when Mg^2+^ ions were incorporated and other reaction parameters were controlled. One can also see that when ACMP/SIM were thrown into SBF solution, Ca^2+^, Mg^2+^ and ${\text{PO}}_{4}^{3}$^−^ in ACMP/SIM partially dissolved and the nanocomposites aggregated linearly into discontinuous networks. About 0.5 h later, Ca^2+^ and ${\text{PO}}_{4}^{3}$^−^ ions redeposited and assembled into HAP nanoplate bundles.

### *In vitro* cytocompatibility and bioactivity of ACMP/SIM

**Figure 4.**
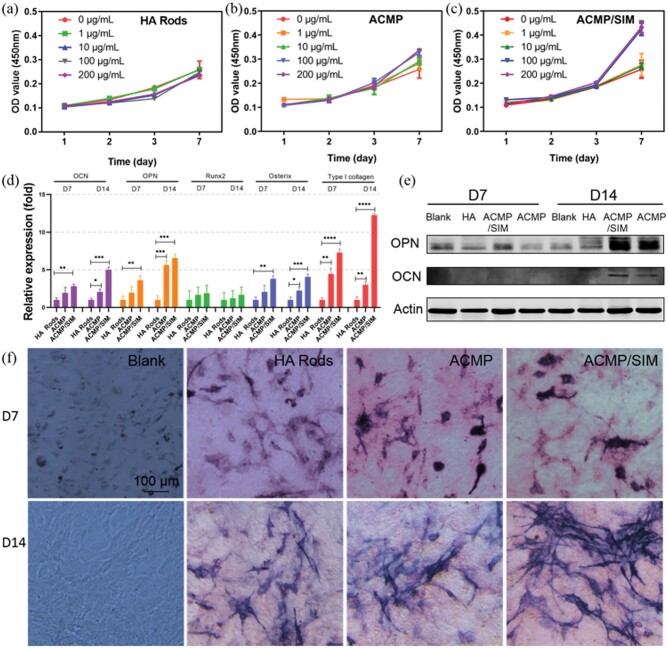
*In vitro* evaluation of the cytocompatibility and bioactivity of ACMP/SIM. The effects of ACMP/SIM nanocomposites and control samples on the proliferation and osteogenic differentiation of MC3T3-E1 osteoblast cells *in vitro*. The proliferation curves of MC3T3-E1s co-cultured with (**a**) HA rods, (**b**) ACMP and (**c**) ACMP/SIM nanoparticles with different concentrations; (**d**) The expression of osteocalcin (OCN), osteopontin (OPN), Runx2, osterix and type I collagen genes of MC3T3-E1s co-cultured with HA rods, ACMP and ACMP/SIM for 7 and 14 days; (**e**) the Western blot assay for the characterization of osteogenic differentiation related proteins including OCN and OPN expressed in co-cultured cells; (**f**) the optical images of ALP in blank and HA rods, ACMP, ACMP/SIM treated MC3T3-E1s after staining (the concentration of the materials used for osteogenic differentiation tests were 100 μg mL^−1^)

Figure 4a–c shows the *in vitro* cytocompatibility of ACMP/SIM nanocomposites and control samples using MC3T3-E1s cells. The results show that ACMP and ACMP/SIM have high cytocompatibility and even can promote the proliferation of MC3T3-E1s within the concentration of 0–200 μg ml^−1^. The ion of Mg^2+^ is a widely existed element in human body, and CaP is the main inorganic composition of bone, herein, it is reasonable that ACMP/SIM has great biocompatibility [2, 40–42]. Then, the effects of ACMP/SIM on osteogenic differentiation of MC3T3-E1s were further investigated. The expression of five osteogenic related genes have been analyzed via RT-PCR (Fig. 4d). The results indicate that total RNA of OCN, OPN, Runx2, Osterix and Type I Collagen are up-regulated, while the ACMP/SIM treated MC3T3-E1s express more osteogenic related genes. The expression of type I collagen genes in ACMP/SIM treated MC3T3-E1s is even about 12 times and 3 times as much as that in the MC3T3-E1s co-cultured with the HA Rods and ACMP for 14 days, respectively. The osteogenic protein expression and ALP of the sample-treated MC3T3-E1s have been also investigated (Fig. 4e, f), the ACMP/SIM treated MC3T3-E1 expressed more OCN, OPN and ALP than the control samples after 7 and 14 days culture. Hence, the above results obviously display the superior bioactivity of ACMP/SIM in promoting osteogenic differentiation of MC3T3-E1s cells.

### *In vivo* performance of ACMP/SIM in bone regeneration

**Figure 5.**
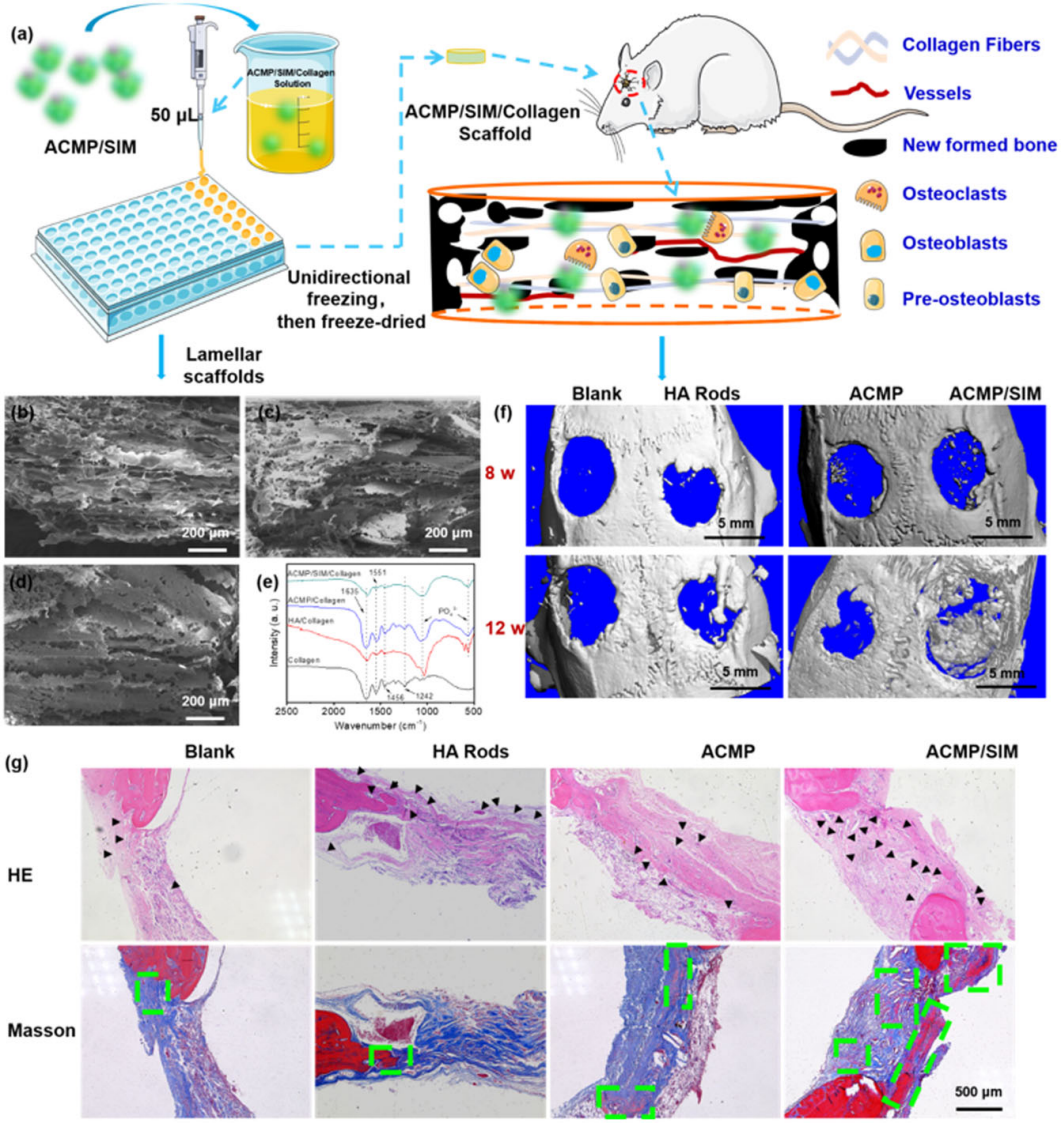
The preparation and *in vivo* performance of lamellar ACMP/SIM/collagen scaffold in the repair of calvaria defect. (**a**) Schematic illustration of the preparation of ACMP/SIM/collagen scaffold and *in vivo* tests of ACMP/SIM/collagen scaffold using a rat defect model with a diameter of 5 mm in calvaria; (**b–d**) SEM images of freeze-dried HA rods/collagen (b), ACMP/collagen (c) and ACMP/SIM/collagen (d) scaffold; (**e**) FTIR spectra of collagen, HA rods/collagen, ACMP/collagen and ACMP/SIM/collagen scaffolds; (**f**) Micro-CT images of the defects (5 mm) in both sides of the calvaria of the rats 8 and 12 weeks post-op; (**g**) the optical images of histological HE and Masson staining of the tissues from the defected part 12 weeks post-op (black arrows: in-growth vessels; green dash rectangles: mature bones)

The *in vivo* performance of ACMP/SIM in promoting bone regeneration has also been investigated using a rat defect model in calvaria with a diameter of 5 mm, and the schematic illustration of the tests is shown in Fig. 5a. First, the ACMP/SIM scaffold with porous structure is necessary to be prepared for the *in vivo* experiments, because the morphology of ACMP/SIM is powder which hard to be directly implanted in defect tissues. It's reported that porous collagen scaffolds with channel-like pore architecture could control cell migration, align ECM fibers along the bone axis, and thereby support cell recruitment and promote directional tissue maturation [37, 43]. Thereby, an ACMP/SIM/Collagen scaffold with aligned channel-like pore has been achieved by controllable freeze-drying method. As shown in Fig. 5b–d, the hybrid scaffolds display channel-like structures with aligned pores. The interconnection and porosity of the scaffold is crucial for diffusion of nutrients and cells, vessels and tissue ingrowths. The FTIR spectra have been used to analysis the chemical composition of the hybrid scaffolds. The absorption peaks at about 1049 and 567 cm^−1^ which are indexed to ${\text{PO}}_{4}^{3}$^−^ groups in the spectra of HA Rods/Collagen, ACMP/Collagen and ACMP/SIM/Collagen clearly indicate the successful integration of the inorganic powders with collagen.

When the ACMP/SIM/Collagen scaffolds were implanted in the animal, no significant side effects were found, and no animal died due to the implanted material. The following results of Micro-CT and histological testing also illustrate the high biosafety and positive effects of the scaffolds. Micro-CT has used to evaluate the *in vivo* performance of the samples. The Micro-CT images shown in Fig. 5f and morphometric analysis of BV/TV and Tb.Th in Supplementary Fig. S6 indicate that ACMP/SIM/Collagen scaffolds are effective in promoting the regeneration of calvaria bone defect 8 and 12 weeks after the surgery, the osteogenic ability of ACMP was greatly enhanced after the incorporation with SIM, and ACMP/SIM/Collagen scaffolds showed a little superiority than HA rods/Collagen scaffolds, but not obvious. Furthermore, hematoxylin-eosin (HE) and Masson’s trichrome staining have been performed to show the new formed bone tissues (Fig. 5g). ACMP/SIM/Collagen group have more blood vessels grown in the bone-defect area, this might be attributed to SIM, which is an effective agent to enhance the angiogenic process [15, 44]. Meanwhile, there are more mature bones uniformly distributed throughout the repair tissues in ACMP/SIM/Collagen group at 12 weeks post-op. The optical images of HE and Masson staining of the tissues from the defected part 8 weeks post-op were given in Supplementary Fig. S7, ACMP/SIM/Collagen group also displayed more newly formed bones. The implanted ACMP/SIM/Collagen scaffolds with highly aligned porous structures could provide sufficient spaces for cells and vessels to grow in. The released Mg^2+^ ions and SIM could promote the osteogenic differentiation of pre-osteoblasts and vessels growing in, respectively. Herein, the combination of ACMP and SIM up-regulated the regeneration of calvaria defect.

Here we show, Mg^2+^ ions played a critical role on the formation of ACMP nanocomposites, ACMP/SIM also has superior cytocompatibility and osteogenic activity due to the corporate effect of ACMP and SIM. ACMP/SIM/Collagen scaffolds with aligned pore structures showed a positive role for bone regeneration using a rat calvaria defect with a diameter of 5 mm. This paper provided a comprehensive study for the development and evaluation of osteogenic biomaterials, which also laid a profound foundation for their translation from the bench to the clinic.

## Conclusions

In this study, amorphous ACMP with different Ca/Mg ratios and ACMP/SIM have been prepared via a simple co-precipitation strategy. The results indicated that Mg^2+^ ions played a critical role in the formation of ACMP by substituting the position of Ca atoms. ACMP/SIM could release Ca^2+^, Mg^2+^ and ${\text{PO}}_{4}^{3}$^−^ when it was immersed into SBF at initial time and transform into HAP phase with a structure of assembled bundles of nanoplates 3 h later. Furthermore, the *in vitro* evaluation of ACMP/SIM was investigated with MC3T3-E1 cells, indicating that ACMP/SIM have high cytocompatibility and could promote the proliferation and osteogenic differentiation of MC3T3-E1s at given concentrations. The superior bioactivity of ACMP/SIM could be explained by the addition of Mg^2+^ ions and SIM. Mg^2+^ ions can stimulate the osteogenic differentiation and enhance bone regeneration, and *in vitro* experiments proved that SIM played a role in promoting osteoblast differentiation. Finally, the *in vivo* performance of ACMP/SIM was also explored using a rat defect model in calvaria with a diameter of 5 mm. A positive effect in promoting regeneration of calvaria defect at 12 weeks post-op was observed, and the combination of ACMP and SIM clearly up-regulated the bone regeneration. These experimental results display an *in vitro* and *invivo* evidence for the effect of ACMP/SIM on bone regeneration and provide a potential strategy for the preparation of bioactive materials for biomedical applications.

## Supplementary data

Supplementary data are available at *REGBIO* online.

## Author contributions

Y.J. gave the conceptualization, conducted most of the experiments and drafted the manuscript; S.T. did the biological experiments and wrote the manuscript; J.H., X.C., Y.L., Z.Z., J.L. and Y.F. helped with the animal experiments; Z.Z. and X.W. helped with the Micro-CT analysis; Q.Y. collected the XANES data, L.L. helped with the analysis of XANES data, participated in the conceptualization and revised the manuscript; and F.C. and S.H. participated in the conceptualization and revised the manuscript.

## Supplementary Material

rbab068_Supplementary_Data
