# Supplementary material for: Amorphous calcium magnesium phosphate nanocomposites with superior osteogenic activity for bone regeneration
Source: Regen Biomater. 2021 Nov 24;8(6):rbab068. doi: 10.1093/rb/rbab068 (PMC8670301; doi:10.1093/rb/rbab068)
Supplement: rbab068_Supplementary_Data [file rbab068_supplementary_data.docx]

**Supporting Information**

**Amorphous Calcium Magnesium Phosphate Nanocomposites with Superior Osteogenic Activity for Bone Regeneration**

*Yingying Jiang,^a,b, †^ Shuo Tan, ^a ,†^ Jianping Hu,^a^ Xin Chen,^a^ Feng Chen,^a,c,^* Qianting Yao,^d^ Xiansong Wang,^e^ Zifei Zhou,^a^ Yunshan Fan,^a^ Junjian Liu,^a^ Yize Lin,^a^ Lijia Liu,^d,^* and Shisheng He,^a,^**

*a Department of Orthopedic, Spinal Pain Research Institute, Shanghai Tenth People’s Hospital, Tongji University School of Medicine, Shanghai 200072, P. R. China, email: jjliu@tongji.edu.cn, tjhss7418@tongji.edu.cn*

*b* Institute of Translational Medicine, Shanghai University, Shanghai, China, 200444*, P. R. China*

*c National Engineering Research Center for Nanotechnology, Shanghai, 200241, P. R. China*

*d Institute of Functional Nano & Soft Materials (FUNSOM), Jiangsu Key Laboratory for Carbon-Based Functional Materials & Devices, Soochow University, 215123, P. R. China, email: lijia.liu@uwo.ca*

*e Shanghai Key Laboratory of Tissue Engineering, Shanghai Ninth People's Hospital, Shanghai Jiao Tong University School of Medicine, Shanghai, 200011, P. R. China*

† These authors contributed equally.

Present address: (L. Liu) Department of Chemistry, University of Western Ontario, 1151 Richmond Street, London, ON, Canada, N6A5B7.

**Table and Figures**

**Table S1.** The atomic percentage of Ca, Mg and P in ACMP with different Ca/Mg ratios and in ACMP/SIM

| Ca/Mg  ratio  Atomic (%) | 10:0 | 10:1  (ACMP) | 10:1 (ACMP/SIM) | 5:5 | 0:10 |
| --- | --- | --- | --- | --- | --- |
| Mg | 0 | 7.30 | 7.04 | 37.66 | 71.78 |
| Ca | 54.82 | 47.68 | 47.55 | 23.64 | 0 |
| P | 45.18 | 45.03 | 45.40 | 38.70 | 27.21 |


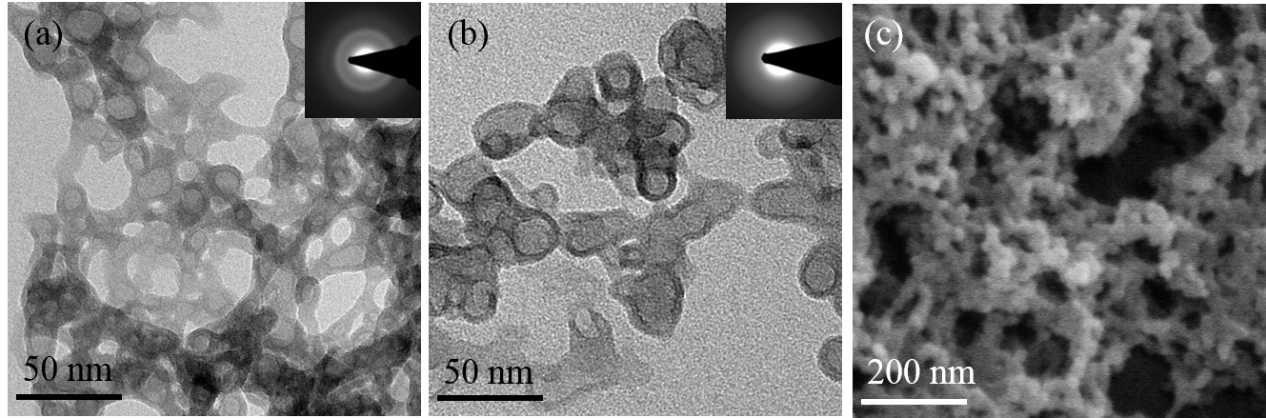


**Fig. S1** TEM micrographs of magnesium incorporated calcium phosphate with a Ca/Mg ratio of (a) 5:5 and (b) 0:10; (c) SEM micrographs of magnesium incorporated calcium phosphate (ACMP) with a Ca/Mg ratio of 10:1.


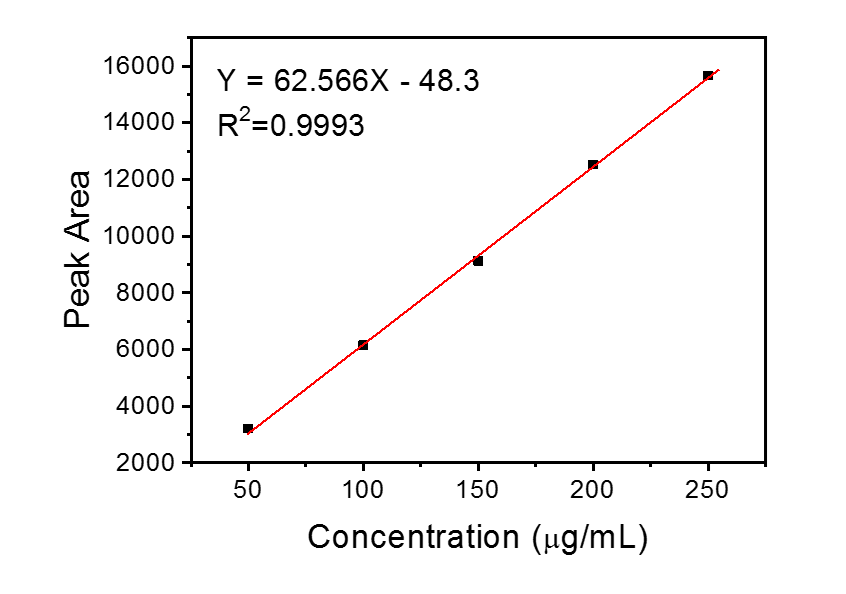


**Fig. S2** The peak area-concentration curve obtained from HPLC chromatogram.


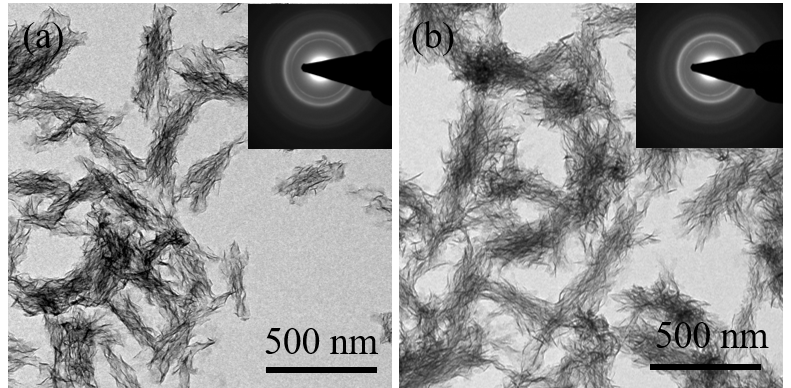


**Fig. S3** TEM images of ACMP/SIM immersed in SBF for (a) 12 h and (b) 36 h (insert: SAED patterns).


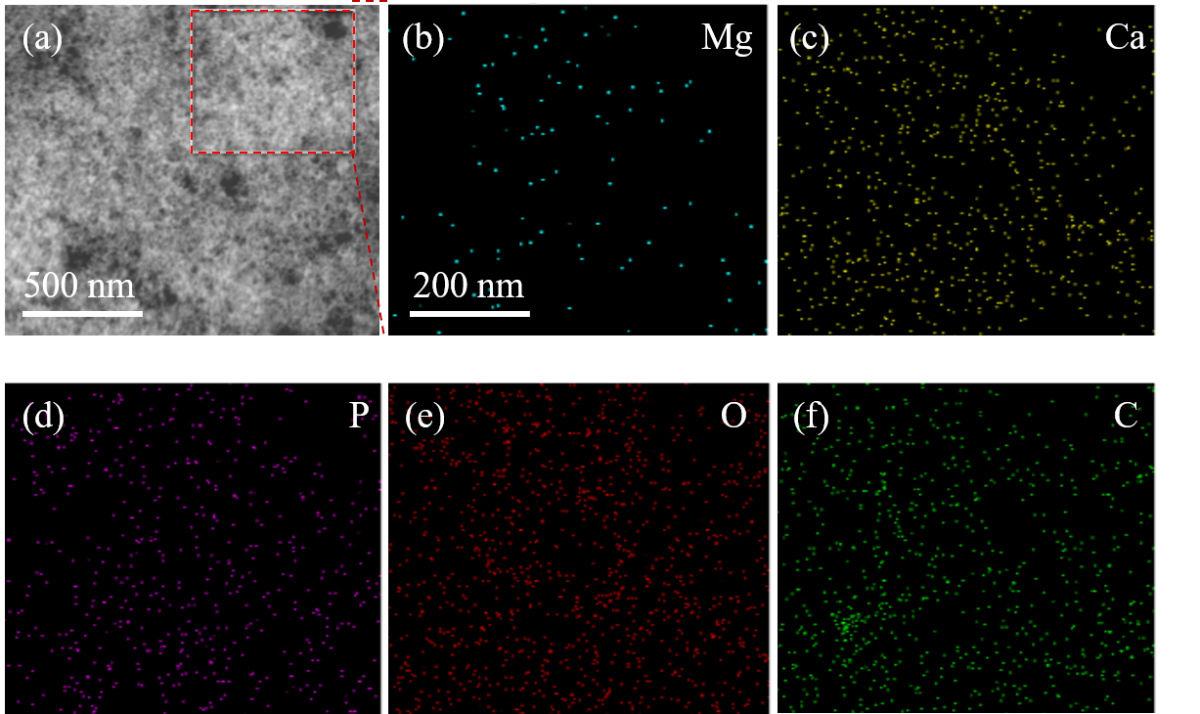


**Fig. S4** (a) STEM images of ACMP/SIM mineralized in SBF for 0.5 h and the Mg (b), Ca (c), P (d), O (e), C (f) distribution mappings of selected area in Fig. S4a.

In Fig. S5a, there is a weaker feature peak “A” and an additional intense peak “B” at the Mg K-edge XANES spectra of ACMP/SIM when compared to Mg_3_(PO_4_)_2_ standards, which reveals the structural differences. While XANES spectra at the Mg K-edge of ACMP/SIM samples in SBF didn’t show obvious changes with time.

In Fig. 5b - c, four discernible peaks can be found with increasing photon energy at the P K-edge. There isn’t any obvious change among the ACMP/SIM samples before and after mineralizing in SBF. Compared to Mg_3_(PO_4_)_2_ standards, ACMP/SIM got a principal peak ‘‘A’’ at lower energy and an obvious post shoulder peak “B”, which result from P 1s to 3p transitions and distortion from Td symmetry[28, 29], respectively. The results indicate that Mg^2+^ plays a critical role in the formation of ACMP by influencing the 1s to 3p transitions of P.


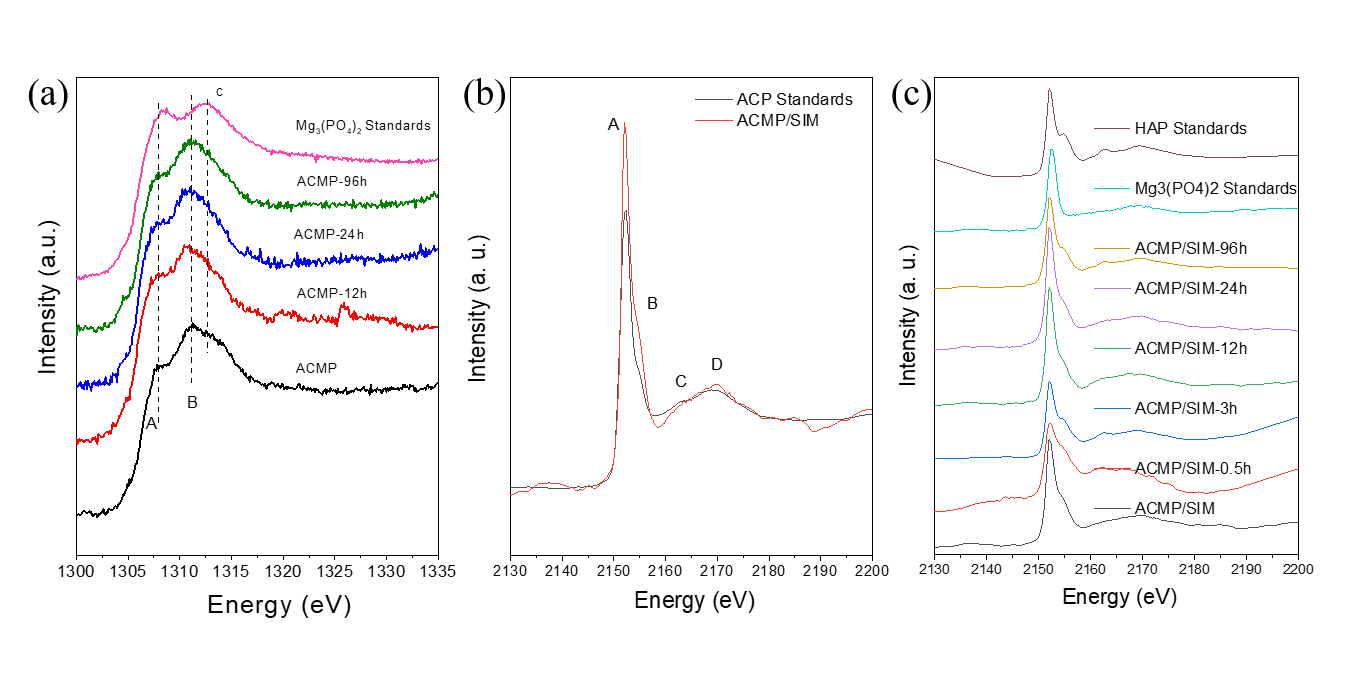


**Fig. S5** (a) Mg K-edge XANES spectra of the ACMP/SIM samples dispersed in SBF for different time and Mg_3_(PO_4_)_2_ standards; (b) P K-edge XANES spectra of ACP standards and and ACMP/SIM; (c) P K-edge XANES spectra of HAP standards, Mg_3_(PO_4_)_2_ standards, and ACMP/SIM samples dispersed in SBF for different time. In (b) and (c), four discernible peaks can be found with increasing photon energy at the P K-edge. There isn’t any obvious change among the ACMP/SIM samples before and after mineralizing in SBF.


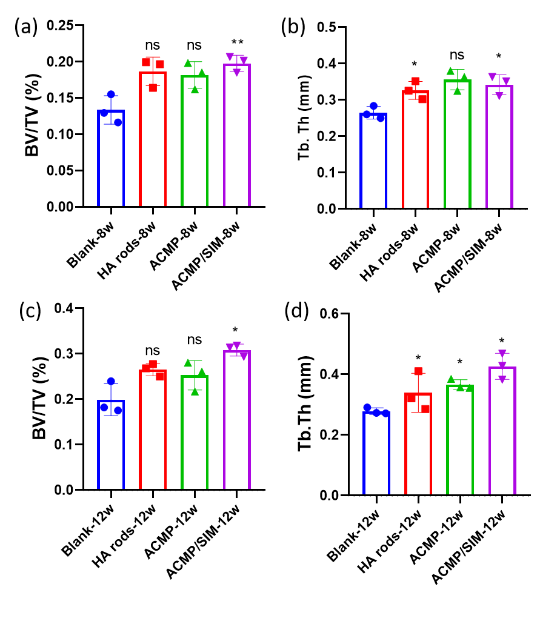


**Fig. S6** The morphometric analysis of BV/TV (a) and Tb.Th (b) for each group at 8 and 12 weeks post operation. (*p < 0.05)


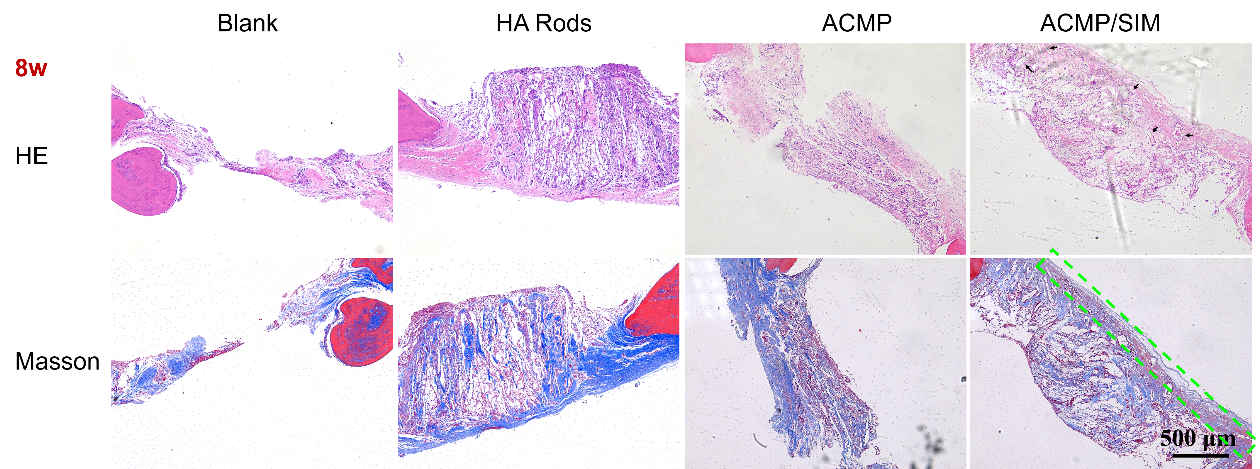


**Fig.** **S7** Optical images of HE and Masson staining of the tissues from the defected part 8 weeks post-op (Black arrows: in-growth vessels; green dash rectangles: mature bones).
